# Patients’ Experience With Enhanced Recovery After Cardiac Surgery

**DOI:** 10.1093/icvts/ivag051

**Published:** 2026-03-20

**Authors:** Evangelos Anastasakis, Krishna Mani, Alexander Smith, Chrysoula Nana, Philemon Gukop, Adnan Charaf, Justin Nowell, Robin Kanagasabay, Marjan Jahangiri

**Affiliations:** Department of Cardiothoracic Surgery, St. George’s Hospital, London SW17 0QT, United Kingdom; Department of Cardiothoracic Surgery, St. George’s Hospital, London SW17 0QT, United Kingdom; Department of Cardiothoracic Surgery, St. George’s Hospital, London SW17 0QT, United Kingdom; Department of Cardiothoracic Surgery, St. George’s Hospital, London SW17 0QT, United Kingdom; Department of Cardiothoracic Surgery, St. George’s Hospital, London SW17 0QT, United Kingdom; Department of Cardiothoracic Surgery, St. George’s Hospital, London SW17 0QT, United Kingdom; Department of Cardiothoracic Surgery, St. George’s Hospital, London SW17 0QT, United Kingdom; Department of Cardiothoracic Surgery, St. George’s Hospital, London SW17 0QT, United Kingdom; Department of Cardiothoracic Surgery, St. George’s Hospital, London SW17 0QT, United Kingdom

**Keywords:** enhanced recovery after surgery, ERAS, cardiac surgery, perioperative care

## Abstract

**Objectives:**

Enhanced recovery after surgery (ERAS) protocols in cardiac surgery can reduce length of stay and incidence of complications, but their impact on patients’ experience is less known. We assessed patients’ perioperative experience with cardiac ERAS.

**Methods:**

We conducted a prospective, single-centre study on 191 consecutive patients undergoing cardiac surgery with ERAS. Questionnaires were administered preoperatively, at 6 weeks and 6 months postoperatively, assessing preoperative concerns, sources of information, education and prehabilitation, expectations vs reality for postoperative recovery, surgical satisfaction, and quality of life.

**Results:**

Preoperatively, pain was the most frequently reported concern. Face-to-face discussions with the surgical team were the most useful source of information, while use of online resources was low. From pre-assessment to surgery, alcohol unit consumption decreased 3-fold. Patients’ median estimations for perioperative risks were mortality 3%, heart attack 5%, stroke 5%, wound infection 10%, and bleeding 5%. Actual pain scores for the first postoperative week were lower than expectations by 1 point on the Numeric Pain Scale, and surgical satisfaction was highest for in-hospital pain control. For the first postoperative month, patients expected to be functionally independent but found they required support with at least 1 activity of daily living. Physical health T-scores increased by 3.1 points at 6 months compared to baseline, whereas mental health scores increased by 2.5 points, meeting and approaching the minimal clinically important difference of 3, respectively.

**Conclusions:**

Our cardiac ERAS protocol facilitates systematic prehabilitation and education. Improved counselling regarding perioperative risks, pain, and recovery may further reduce perioperative stress.

**Clinical registration number:**

St. George’s Hospital R&D Ref: AU0035.

## Graphical abstract

**Figure:**
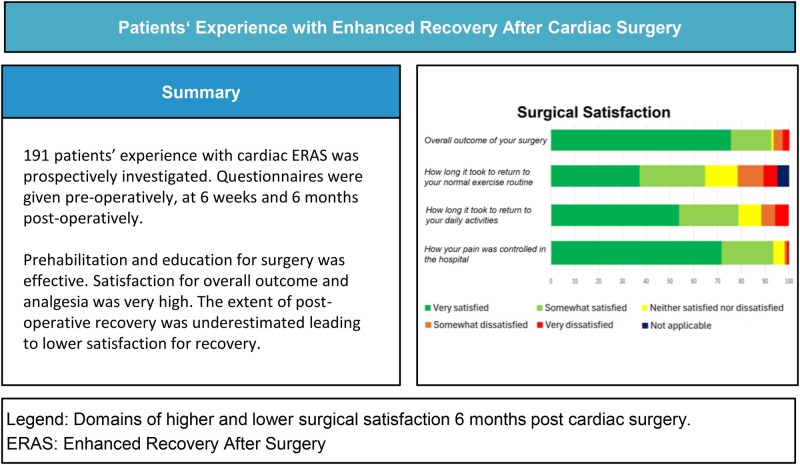

## INTRODUCTION

In recent years, low mortality for cardiac surgery has been reported in national databases.^1^ Less reported are the patient’s perception of complications and patient-reported outcomes including quality of life (QOL). These are important for shared decision-making, given percutaneous alternatives are being offered to patients for some conditions. There has been a shift towards more minimally invasive options and faster recovery.

The cardiac enhanced recovery after surgery (ERAS) guidelines were first published in 2019 and recently updated in 2024.^2^^,^^3^ A recent systematic review and meta-analysis investigating cardiac ERAS protocols shows they can reduce the length of hospital stay and incidence of postoperative complications.^4^

ERAS protocols are delivered by multi-disciplinary teams (MDTs), placing the patient at the forefront to improve postoperative outcomes. However, to our knowledge, no studies analyse patients’ experience with cardiac ERAS protocols.

## PATIENTS AND METHODS

### Study design

This was a prospective, single-centre, observational study. Local ethical approval was obtained (Ref: AU0035). The study was performed in accordance with the ethical standards as laid down in the 1964 Declaration of Helsinki and its later amendments. Any collection and storage of data has been consistent with the WMA Declaration of Taipei. Consecutive patients, >18 years of age, undergoing elective and urgent cardiac surgery between November 2022 and October 2023 were invited. Urgent and elective surgery was defined as per the Society for Cardiothoracic Surgery in Great Britain and Ireland. Elective surgery was defined as routine admission for surgery, whereas urgent surgery was patients who were not admitted electively and could not be discharged without a definitive procedure.^5^ Informed, written consent was obtained. Patients were excluded if they underwent emergency or salvage cardiac surgery; were unable to consent due to inadequate comprehension of English; or refused to participate. Data were collected with questionnaires at 3 timepoints: preoperatively and at 6 weeks and 6 months postoperatively (**Figure S1**). Baseline clinical characteristics, type of operation, and postoperative outcomes were prospectively collected, up to 6 months after surgery.

### Sample size

The sample size was derived from estimations based on a previous questionnaire-based cohort study from our group.^5^ Our unit performs approximately 700 operations annually under 8 primary surgeons. We assumed a minimum annual caseload of 250 cases among the 3 primary surgeons recruiting to this study, of which 60% would meet the inclusion criteria and consent to participate. We arrived at a sample size of 150, which would provide adequate precision for proportions of around 50% (±8% margin of error at a 95% confidence level). We anticipated a loss of follow-up of approximately 20% and set out to recruit 188 patients.

### Survey questionnaire

Questionnaires were designed by a multidisciplinary research team comprising of survey research experts, cardiac surgeons, and patients’ representatives for our pilot study.^6^ Following publication of the pilot study, a new heart research team comprised of cardiac surgeons, cardiac anaesthetists and intensivists, all experts in cardiac ERAS, adapted the pilot questionnaire to be delivered in a prospective manner at 3 timepoints. Questions assessed 5 domains: (1) preoperative concerns; (2) sources of information; (3) education and prehabilitation; (4) expectations vs reality for postoperative recovery; and (5) surgical satisfaction and QOL (see **Supplementary Data** for questionnaires). Questionnaires were trialed on 5 patients, not included in analysis, to ensure questionnaires were an appropriate length and suitable to understand and answer. After making changes based on feedback, the questionnaires were trialed on a further 3 patients.

Surgical satisfaction was measured using a modified surgical satisfaction questionnaire (SSQ-8), including a question for scar appearance.^6^ Independence with activities of daily living (ADLs) was assessed using a modified Katz Index of Independence in ADL.^7^ Pain scores were assessed using the Numeric Pain Rating Scale.^8^ QOL was assessed with the Patient-Reported Outcomes Measurement Information System (PROMIS) global health measure v1.2.^9^ A score of 50 denotes the population mean and the minimal clinically important difference (MCID) was defined as 3 T-score points.^9^

### Clinical management

Our cardiac ERAS protocol (**Table S1**) is derived from the 2019 guidelines and was implemented at our institution in October 2020.^2^ All aspects are audited every 3 months to ensure compliance >70%. Elective patients were seen preoperatively in the clinic by a consultant cardiac surgeon and in pre-assessment clinic by a surgeon and a nurse. At pre-assessment, patients were educated for their operation; provided with the relevant British Heart Foundation (BHF) and local leaflets for their condition and operation; and directed to online patient engagement tools. Patients were prehabilitated by being counselled on alcohol and smoking cessation; advised on nutritional optimization; and identifying nutritional deficiency, anaemia, or diabetes following blood tests. If any abnormalities were identified on blood tests requiring optimization, patients were reviewed in pre-assessment clinic again with an anaesthetist. Pre-frail patients, defined as Rockwood Clinical Frailty Score >3 or patients with pulmonary comorbidities requiring the long-term use of bronchodilators, were identified for referral for controlled exercise programmes. Controlled exercise programmes are delivered face-to-face by the cardiac rehabilitation team preoperatively. These include cardiovascular and strengthening exercises, and incentive spirometry that can be undertaken at home preoperatively. They are seen by the cardiac rehabilitation team weekly. The programme is based on the BHF cardiac rehabilitation exercise programme.

Urgent patients were reviewed by the pre-assessment team as inpatients, and delivered an accelerated preoperative ERAS bundle, which omits controlled exercise programmes.

All urgent and elective patients underwent blood tests, coronary angiography, and transthoracic echocardiography and were discussed at the cardiac MDT. Patients undergoing aortic surgery, also underwent CT and/or MRI aortogram and were discussed at the aortic MDT meeting.

All patients were admitted to intensive care unit (ICU) after surgery. Extubation is aimed within 6 hours of ICU arrival. When ICU discharge criteria were met, patients were transferred to the ward (**Table S2**). Multi-modal analgesia is administered to all patients and includes a combination of local anaesthetic or regional block infiltration at the end of surgery, regular paracetamol, the use of dexmedetomidine in the ICU and as required tramadol, pregabalin or gabapentin. Patients are discharged on regular paracetamol with either as required dihydrocodeine or tramadol. Following discharge, patients were reviewed in clinic at 6 weeks postoperatively, either face-to-face or by telephone consultation. At this point, patients were sent the postoperative questionnaire, based on their preferences, by email or post. If no further management or follow-up was necessary, patients were discharged from cardiac surgery to their general practitioner or cardiology.

### Statistical analysis

Statistical analysis was performed from paired data from different timepoints. Each question was analysed individually. Patients were only included in analysis if data were collected at all timepoints for the specific question. Patients with incomplete data were excluded from the specific question, but not from other questions. Continuous data were checked for normality using histograms and QQ-plots. Differences in smoking and alcohol consumption, before and after pre-assessment clinic, were compared using Wilcoxon-matched signed rank test. Preoperative expectations vs reality at 6 weeks postoperatively for key recovery milestones were compared by ordinal conversion of timepoints to numbers and comparison of medians using Wilcoxon-matched signed rank test. Preoperative expectations vs reality for pain scores and independence with ADLs were compared using Wilcoxon-matched signed rank test. QOL was compared using the Friedman test and corrected using Dunn’s multiple comparisons test. Correlation analysis between EuroSCORE II and self-estimated mortality risk was performed using Spearman’s rank correlation. Statistical significance was indicated by a *P-*value <.05. Continuous variables are expressed as mean ± standard deviation and categorical variables as numbers with percentages. Statistical analyses were performed using GraphPad Prism, Version 10.2.3; IBM SPSS Statistics for Windows, Version 29.0.2.0; and Microsoft Excel 2021 software.

## RESULTS

Between November 2022 and October 2023, 191 consecutive patients were recruited. Baseline characteristics are shown in **Table 1**. Operative data are shown in **Table 2**.

**Table 1.** Baseline Characteristics

	All cases *N* = 191 (%)
Age, years	66 (58-74)
EuroSCORE 2, %	1.4 (0.9-2.3)
Male, sex	159 (83.2)
Female, sex	32 (16.8)
Preoperative questionnaire	191 (100)
Early postoperative questionnaire	134 (70.2)
Late postoperative questionnaire	135 (70.7)
Creatinine clearance	
>85 mL/minute	106 (55.5)
50-85 mL/minute	66 (34.6)
<50 mL/minute	16 (8.4)
Dialysis	3 (1.6)
Extracardiac arteriopathy	20 (10.5)
Poor mobility	1 (0.5)
Previous cardiac surgery	6 (3.1)
Chronic lung disease	24 (12.6)
Active endocarditis	5 (2.6)
Diabetes on insulin	24 (12.6)
NYHA	
I	74 (38.7)
II	93 (48.7)
III	21 (11.0)
IV	3 (1.6)
CCS class 4 angina	13 (6.8)
Left ventricular ejection fraction, %	
>50	154 (80.6)
31-50	32 (16.8)
21-30	5 (2.6)
Recent myocardial infarction	13 (6.8)
Surgery on thoracic aorta	33 (17.3)
Smoking	
Non-smoker	95 (49.7)
Ex-smoker	67 (35.1)
Active smoker	29 (15.2)
Comorbidities	
Hypertension	105 (55.0)
Diabetes mellitus	66 (34.6)
Chronic obstructive pulmonary disease	5 (2.6)
History of myocardial infarction	45 (23.6)
History of stroke or TIA	8 (4.2)
Connective tissue disease	2 (1.0)
Hypercholesterolaemia	69 (36.1)
Atrial fibrillation	15 (7.9)
History of alcohol excess	7 (3.7)
Kidney disease	9 (4.7)

Data reported as median (IQR 25%-75%) for continuous variables and number (%) for categorical variables. History of alcohol excess defined as a history >35 units/week for women and >50 units/week for men.

Abbreviations: CCS, Canadian Cardiovascular Society; NYHA, New York Heart Association; TIA, transient ischaemic attack.

**Table 2.** Operative Data

	All cases *N* = 191 (%)
Classification of intervention	
Elective	86 (45.0)
Urgent	105 (55.0)
Redo procedure	6 (3.1)
Isolated procedures	
Aortic surgery	5 (2.6)
CABG	107 (56.0)
Valve surgery	17 (8.9)
Other	9 (4.7)
Concomitant procedures	
Aortic surgery	6 (3.1)
CABG + aortic surgery	5 (2.6)
CABG + valve surgery	18 (9.4)
CABG + aortic surgery + valve surgery	3 (1.6)
CABG + other	3 (1.6)
Valve surgery	1 (0.5)
Valve surgery + aortic surgery	12 (6.3)
Valve surgery + aortic surgery + other	2 (1.0)
Valve surgery + other	2 (1.0)
Other	1 (0.5)
Cross-clamp time, minutes	66 (46-81.25)
Cardiopulmonary bypass time, minutes	91 (70.2-113)

Data reported as median (IQR 25%-75%) for continuous variables and number (%) for categorical variables.

Abbreviation: CABG, coronary artery bypass graft surgery.

Thirty-day mortality was 1 (0.5%) patient. Four (2.1%) patients developed transient ischaemic attack or stroke, 3 (1.6%) required intra-aortic balloon pump insertion, 6 (3.1%) required resternotomy for bleeding, 1 (0.5%) required tracheostomy insertion, and 7 (3.7%) required haemofiltration. Median ICU length of stay was 1.5 (1-2.5) days, and hospital length of stay was 6 (5-9) days. Ten (5.2%) were re-admitted to ICU.

Mortality at 6 months was 7 (3.7%) patients. Permanent pacemakers were inserted in 5 (2.6%) patients, 2 (1%) required redo surgery, 14 (7.3%) developed a superficial skin infection, and 3 (1.6%) developed a myocardial infarction. Twenty-nine (15.2%) were re-admitted to hospital.

### Preoperative concerns

**Figure 1.**
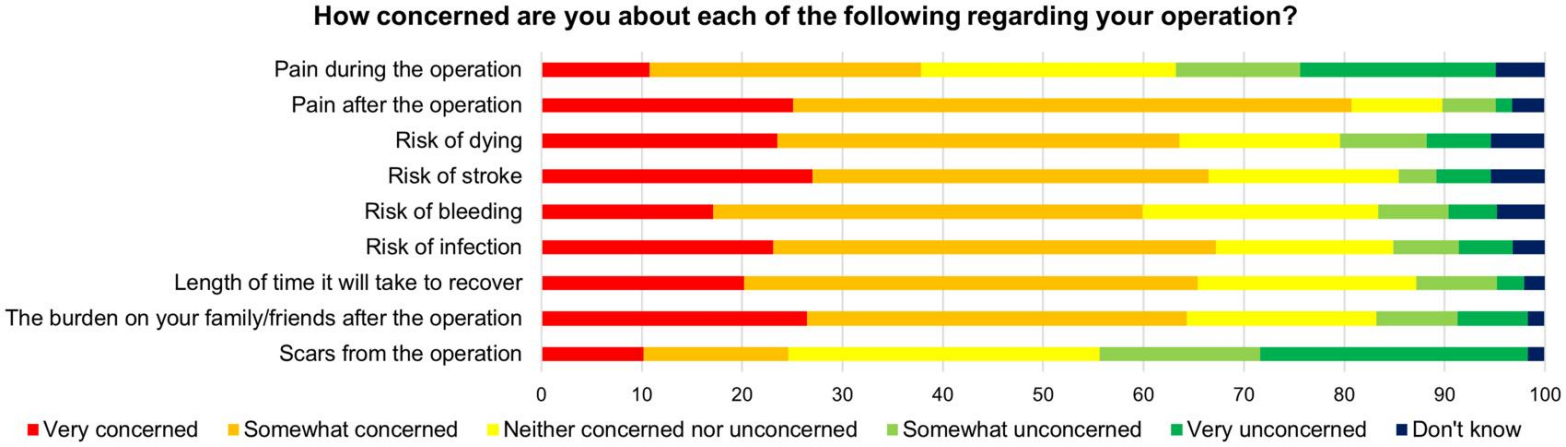
Proportional Responses for Domains of Preoperative Concerns (*N* = 178).

Domains of concern preoperatively for cardiac surgery are shown in **Figure 1**.

### Sources of information

**Figure 2.**
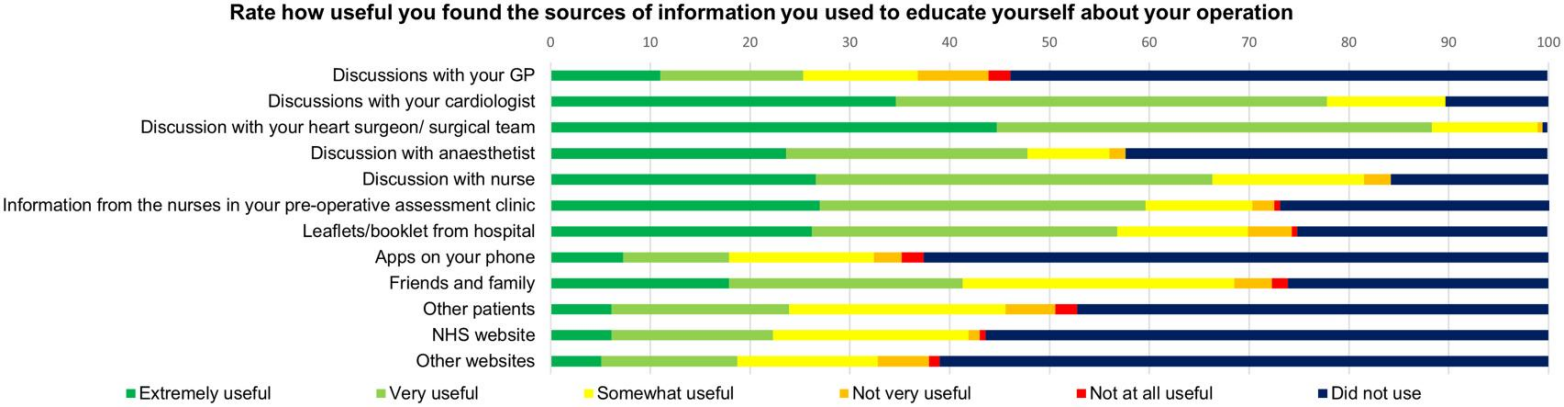
Proportions of Usefulness of Sources of Information Used Preoperatively by Patients for Education About Cardiac Surgery (*N* = 191).

Usefulness of preoperative sources of information is shown in **Figure 2**.

### Education and prehabilitation

Patients’ estimations of perioperative complications were mortality 3% (1-5), bleeding 5% (2-10), wound infection 10% (3-15), heart attack 5% (1-10), and stroke 5% (1.6-10) (*N* = 186). Correlation analysis shows weak but statistically significant positive association between EuroSCORE II and patient-estimated mortality (ρ = 0.248, *P* = .001; *N* = 186).

**Figure 3.**
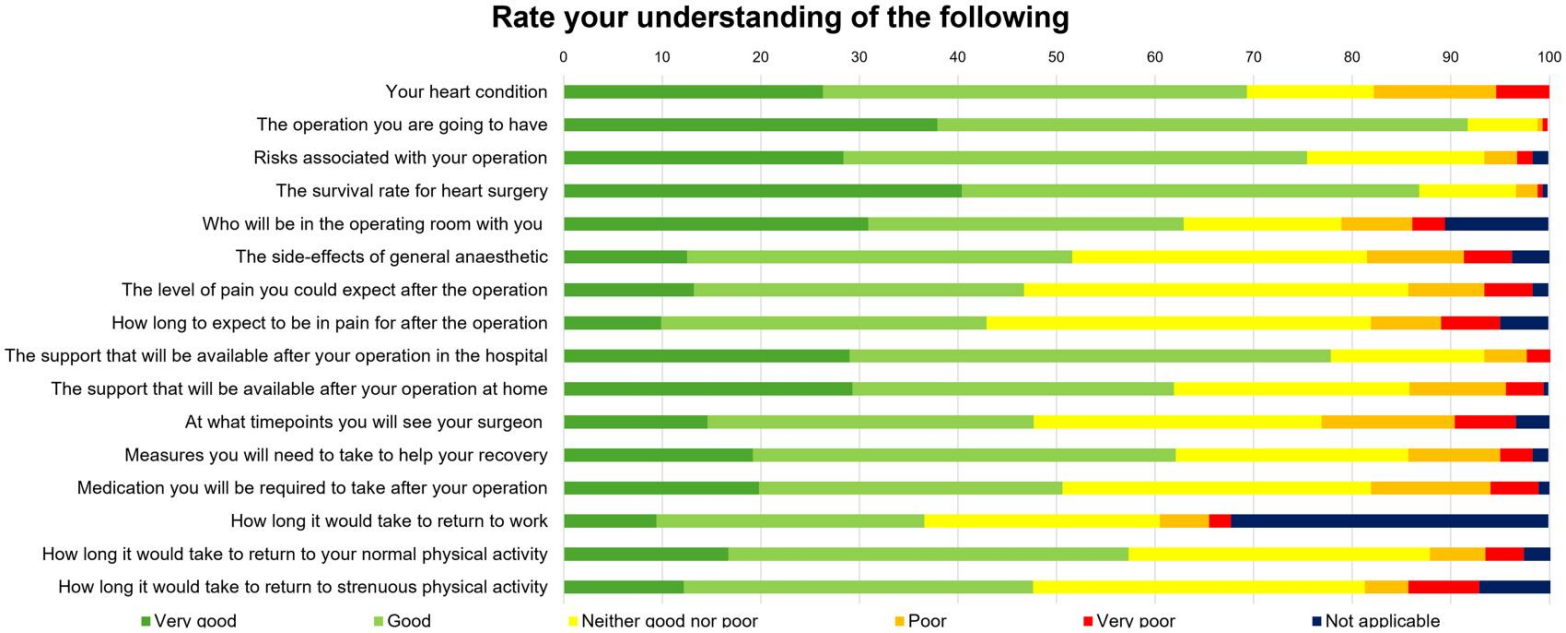
Proportional Preoperative Levels of Understanding of Cardiac Surgery, Risks, and Recovery (*N* = 186).

Proportional levels of preoperative understanding are shown in **Figure 3**.

In smokers, daily cigarette consumption significantly decreased from pre-assessment to surgery: 10 (5-20) vs 5 (0-8) (*P* < .01; *N* = 14). Weekly alcohol consumption significantly decreased from pre-assessment clinic to surgery: 3 (0-11) vs 1 (0-7.5) (*P* < .01; *N* = 89) (**Figure S2**).

### Expectations vs reality for postoperative recovery

**Figure 4.**
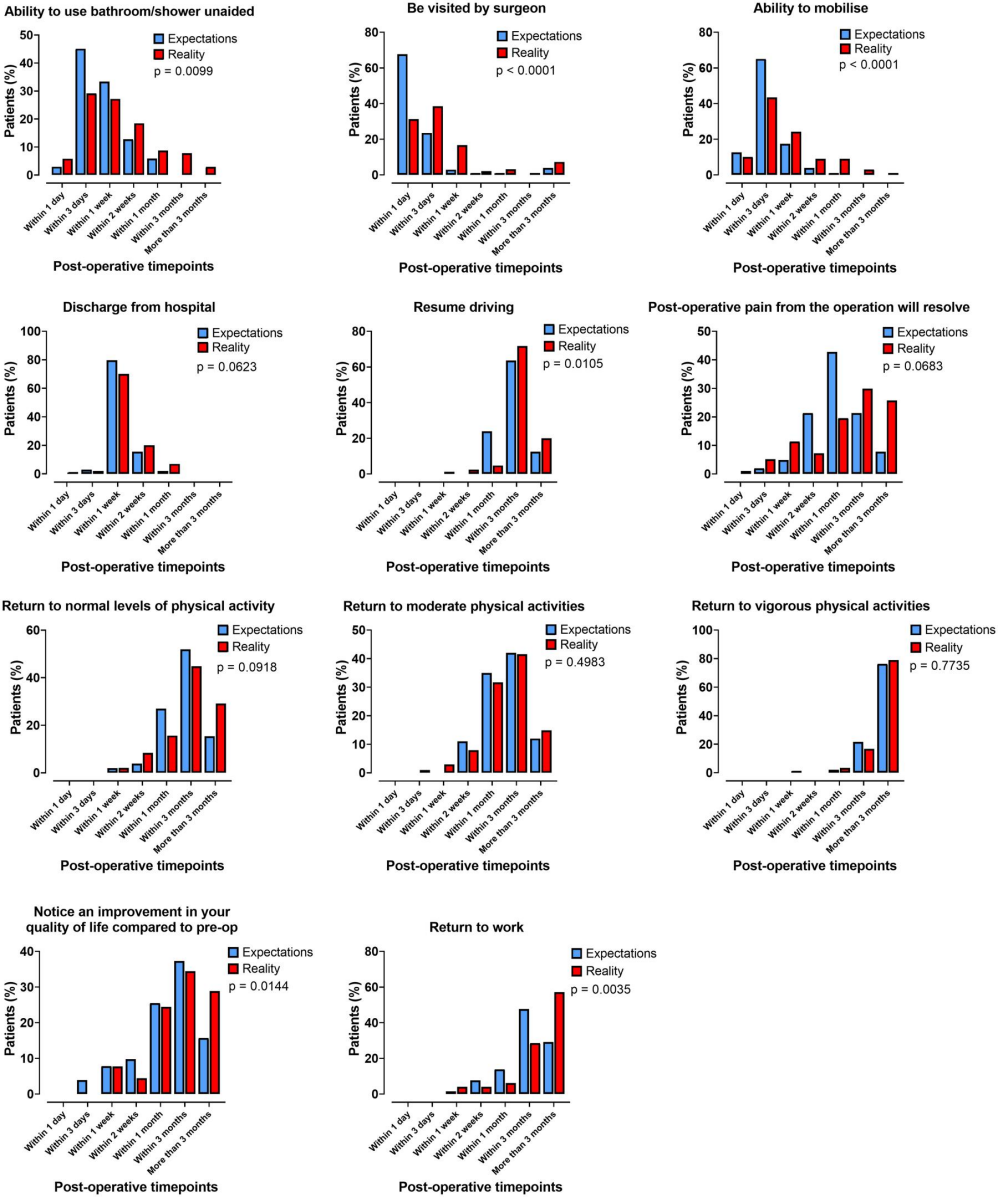
Expectations vs Reality for Key Milestones of Postoperative Recovery. *P* is the value for comparison of postoperative timepoints using Wilcoxon-matched-pairs signed rank test to compare between the 2 groups (*N* = 125).

Expectations vs reality for key recovery milestones are shown in **Figure 4**.

Patients significantly overestimated pain scores in the first postoperative week: expectations 7 (5-8) vs reality 6 (4-8) (*P* < .01; *N* = 128). There was no difference in expectations vs reality for pain scores between 1 week and 1 month postoperatively: expectation 4 (3-5) vs reality 4 (2-6) (*P* = .95; *N* = 128).

There was no difference in expectation vs reality for support required with ADLs in the first week after discharge: expectation 2 (0-4) vs reality 2 (1-4) (*P* = .21; *N* = 126). Patients significantly underestimated the number of ADLs they would need support with between 1 week and 1 month after discharge: expectation 0 (0-2) vs reality 0 (1-2.5) (*P* < .01; *N* = 126).

### Surgical satisfaction and QOL

**Figure 5.**
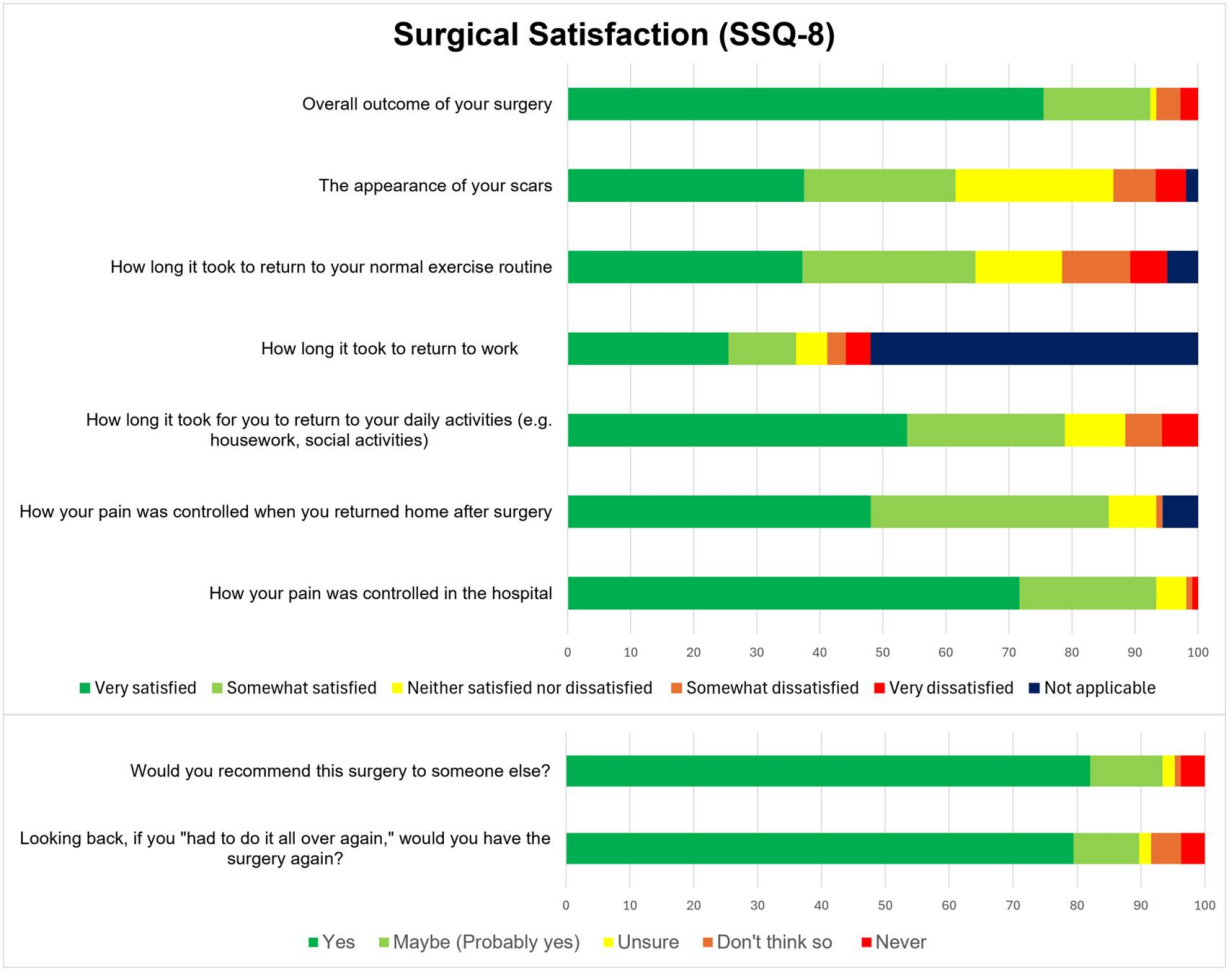
Surgical Satisfaction Assessed by the Surgical Satisfaction Questionnaire (SSQ-8) at 6 Months Postoperatively (*N* = 135).

Surgical satisfaction (SSQ-8) at 6 months postoperatively is shown in **Figure 5**.

For QOL, physical health T-score was 47.70 (42.30-54.10) at baseline; 47.70 (44.90-55) at 6 weeks; and 50.80 (44.90-57.70) at 6 months postoperatively. Physical health significantly improved at 6 months compared to baseline and this met the MCID (*P* = .02; *N* = 109). There was no change when comparing baseline to 6 weeks (*P* = .53; *N* = 109) and 6 weeks to 6 months (*P* = .53; *N* = 109).

Mental health T-score was 50.80 (48.30-56) at baseline; 50.80 (45.80-56) at 6 weeks; and 53.30 (48.30-59) at 6 months. There was no statistically significant change in mental health when comparing baseline to 6 weeks (*P* > .99, *N* = 109); baseline to 6 months (*P* = .21, *N* = 109); and 6 weeks to 6 months (*P* = .19, *N* = 109). The change between baseline and 6 months approached the MCID (**Figures S3**  **and**  **S4**).

## DISCUSSION

In this study, we have assessed patients’ preoperative education and prehabilitation; sources of information; and identified areas of preoperative knowledge, which correlate with preoperative concerns, postoperative recovery, and surgical satisfaction.

Prehabilitation offers patients the opportunity to eliminate life-long, high-risk behaviours.^10^ Such examples are alcohol and smoking. Our pre-assessment team effectively reduced patients’ alcohol and smoking consumption for surgery.

Our cohort, with a median age of 66, preferred face-to-face education by their surgical team, with a low uptake of online resources. Patient engagement tools including applications, online resources, and printed materials are advocated in cardiac ERAS guidelines.^2^ Mobile applications are being increasingly developed and may be more suited to future generations undergoing cardiac surgery.^11^ It is important to tailor preoperative education as effective delivery can significantly reduce length of ICU stay; pre- and postoperative anxiety and depression; and can improve patient satisfaction.^12^^,^^13^ Furthermore, preoperative anxiety is a risk factor for major morbidity and mortality and positively correlates with higher postoperative pain scores and analgesic demand.^14–17^

Our cohort slightly overestimated the risks associated with surgery. They quoted a mortality of 3%, when the cohort’s EuroSCORE II was 1.7%. Following CABG, the National Adult Cardiac Surgery Audit (NACSA) quotes the incidence of resternotomy for bleeding as 1.2% and stroke or transient ischaemic attack 0.8%, which are lower than our cohort’s estimations.^18^ Estimations for postoperative myocardial infarction and wound infection were more accurate.^18^^,^^19^ Postoperative complications were significant sources of preoperative concern and better education of contemporary complication rates can reduce anxiety.

Patients were most concerned about postoperative pain. Moreover, they had low levels of understanding for intensity and duration of postoperative pain. This is an unnecessary source of stress, given the cohort’s pain score in the first postoperative week was significantly lower than expectations and surgical satisfaction was highest for in-hospital pain control. This highlights the effectiveness of multimodal, opioid sparing analgesia in cardiac ERAS.^2^

Patients were also concerned about the recovery process following cardiac surgery. There were low levels of understanding for postoperative pain, support at home, follow-up, medications, and return to physical activities. Their expectations for recovery significantly underestimated multiple milestones. Patients significantly underestimated support with ADLs between 1 week and 1 month postoperatively. Consequently, satisfaction was lower for return to physical activities and work.

Large studies in multiple surgical specialties have shown that fulfilment of preoperative expectations predicts postoperative satisfaction, correlates with QOL and improves patient reported outcome measures.^20–22^ Studies in cardiac surgery have shown that negative preoperative expectations are associated with prolonged recovery and increased postoperative disability; decreased QOL; and higher postoperative anxiety and depression.^23^^,^^24^

The manipulation of preoperative expectations using psychological interventions has emerged as a therapeutic target for cardiac surgery.^25^ The PSY-HEART trial, a single-centre, 3-arm randomized controlled trial (RCT) has shown that psychological interventions significantly improve disability at 6 months postoperatively and reduce serum concentrations of pro-inflammatory interleukin-6 and interleukin-8.^26^ The ValvEx study, a multicentre RCT, showed that expectation-focused intervention may optimize outcomes after valve surgery for patients with a high need for information.^27^ The protocol of PSY-HEART II, another multicentre RCTs, has been published and will further inform the use of psychological interventions for cardiac surgery.^28^

There was high surgical satisfaction for overall outcome. This correlates with the observed improvements in physical health. While surgical satisfaction was highest for in-hospital pain control, satisfaction with pain control after discharge was lower. This can be highlighted for future iterations of cardiac ERAS.

Surgical satisfaction was lowest for scar appearance and length of time to return to daily activities and exercise routine. Improved satisfaction in the above domains may be achieved with minimally invasive surgery. This is commonly incorporated in ERAS guidelines.^3^ However, the updated ERAS consensus statement does not grade recommendations for minimally invasive cardiac surgery, stating future research is required to establish mid- and long-term outcomes.^3^

### Limitations and future directions

The main limitation of this study is the lack of a comparator group without ERAS. As cardiac ERAS is evidence-based, it is unethical to withhold ERAS applications from patients. Our future directions include propensity-score matching historical controls from our group’s previous study investigating QOL and recovery using the Short Form Health Survey (SF-36) and the Postoperative Quality of Recovery Scale (PostopQRS).^5^

Furthermore, not all elements of our preoperative cardiac ERAS bundle can be effectively applied to patients undergoing urgent cardiac surgery.

## CONCLUSION

Cardiac ERAS facilitates systematic prehabilitation and education with patients demonstrating significant smoking and alcohol cessation and appropriate understanding of perioperative risk. Patients prefer face-to-face education by the multidisciplinary heart team, while the use of digital resources is low. There is high postoperative satisfaction for overall outcome and pain control. Thorough education about risks and postoperative pain and managing expectations for recovery have been identified as means to reduce perioperative stress and further improve satisfaction.

## Supplementary Material

ivag051_Supplementary_Data

## Data Availability

The data underlying this article will be shared on reasonable request to the corresponding author.
