# Supplementary material for: Patients’ Experience With Enhanced Recovery After Cardiac Surgery
Source: Interdiscip Cardiovasc Thorac Surg. 2026 Mar 20;41(3):ivag051. doi: 10.1093/icvts/ivag051 (PMC13006205; doi:10.1093/icvts/ivag051)
Supplement: ivag051_Supplementary_Data [file ivag051_supplementary_data.zip › Supplementary material 2 - study questionnaires.pdf]

St George's University of London — St George's University Hospitals NHS Foundation Trust

Pre-operative preparation, concerns and expectations of cardiac surgery

Thank you for taking part in this survey. We would like to reassure you that all answers are anonymous.

The purpose of this survey is to help improve the care of patients undergoing heart surgery by exploring your experience.

You will be asked to complete 3 questionnaires. The first is in the pre-operative phase. The second will be in the early post-operative phase. The third will be in the late post-operative phase.

This is the pre-operative questionnaire.

In this survey we would like to find out what is concerning you and the effects you expect the surgery to have on your quality of life.

This survey should take approximately 10-15 minutes to complete

Hospital Number

Gender

○ Male
○ Female

Date of Birth – dd/mm/yyyy

/ /
____

Date completing survey - dd/mm/yyyy

/ /
____

Estimated date of operation - dd/mm/yyyy

/ /
____

Would you like to receive the 2 post-operative questionnaires via post or email? (If you select email please provide your email address on the black line)

○ Post
○ Email: ______________________________

1. To what extent are you concerned about your upcoming operation?

- ○ Very Concerned
- ○ Somewhat concerned
- ○ Neither concerned nor unconcerned
- ○ Somewhat unconcerned
- ○ Very unconcerned
- ○ Don't know

2. How concerned are you about each of the following regarding your operation?

	Very Concerned	*Somewhat concerned*	*Neither concerned nor unconcerned*	*Somewhat unconcerned*	*Very unconcerned*	*Don't know*
Pain during the operation	○	○	○	○	○	○
Pain after the operation	○	○	○	○	○	○
Risk of dying	○	○	○	○	○	○
Risk of stroke	○	○	○	○	○	○
Risk of bleeding	○	○	○	○	○	○
Risk of infection	○	○	○	○	○	○
The length of time it will take to recover	○	○	○	○	○	○
The burden on your family/friends after the operation	○	○	○	○	○	○
Scars from the operation	○	○	○	○	○	○

3. Is there anything else you are concerned about that has not been mentioned?

4. What do you think is the % risk of the following complications after heart surgery?

Dying	%
Heart attack	%
Stroke	%
Wound infection	%
Bleeding	%

5. **Overall, do you feel prepared for your operation?**

- ◯ Yes
- ◯ Unsure
- ◯ No

6. **If you answered unsure or no, why do you not feel prepared for your operation?**

7. **Rate your understanding of the following:**

	Very good	*Good*	*Neither good nor poor*	*Poor*	*Very poor*	*Not applicable*
Your heart condition	◯	◯	◯	◯	◯	◯
The operation you are going to have	◯	◯	◯	◯	◯	◯
Risks associated with your operation	◯	◯	◯	◯	◯	◯
The survival rate for heart surgery	◯	◯	◯	◯	◯	◯
Who will be in the operating room with you	◯	◯	◯	◯	◯	◯
The side-effects of general anaesthetic	◯	◯	◯	◯	◯	◯
The level of pain you could expect after the operation	◯	◯	◯	◯	◯	◯
How long to expect to be in pain for after the operation	◯	◯	◯	◯	◯	◯
The support that will be available after your operation in the hospital	◯	◯	◯	◯	◯	◯
The support that will be available after your operation at home	◯	◯	◯	◯	◯	◯
At what timepoints you will see your surgeon	◯	◯	◯	◯	◯	◯
Measures you will need to take to help your recovery	◯	◯	◯	◯	◯	◯
Medication you will be required to take after your operation	◯	◯	◯	◯	◯	◯
How long it would take to return to work	◯	◯	◯	◯	◯	◯
How long it would take to return to your normal physical activity	◯	◯	◯	◯	◯	◯
How long it would take to return to strenuous physical activity	◯	◯	◯	◯	◯	◯

8. **Are there any other topics you would like to have been provided information on before your operation?**

9. **Rate how useful you found the sources of information you used to educate yourself about your operation**

	Extremely useful	*Very useful*	*Somewhat useful*	*Not very useful*	*Not at all useful*	*Did not use*
Discussions with your GP	○	○	○	○	○	○
Discussions with your cardiologist	○	○	○	○	○	○
Discussion with your heart surgeon/ surgical team	○	○	○	○	○	○
Discussion with anaesthetist	○	○	○	○	○	○
Discussion with nurse	○	○	○	○	○	○
Discussion with hospital physiotherapist	○	○	○	○	○	○
Discussion with hospital pharmacist	○	○	○	○	○	○
Information from the nurses in your pre-operative assessment clinic	○	○	○	○	○	○
Leaflets/booklet from hospital	○	○	○	○	○	○
Apps on your phone	○	○	○	○	○	○
Friends and family	○	○	○	○	○	○
Other patients	○	○	○	○	○	○
NHS Website	○	○	○	○	○	○
Other websites	○	○	○	○	○	○

10. How satisfied are you with the following?

	Very satisfied	*Somewhat satisfied*	*Neither satisfied nor dissatisfied*	*Somewhat dissatisfied*	*Very dissatisfied*	*Not applica ble*
How often you saw your surgeon/ surgical team	◯	◯	◯	◯	◯	◯
Your ability to contact your surgical team or the hospital to ask any questions	◯	◯	◯	◯	◯	◯
How often you saw your other doctors such as your cardiologist or GP	◯	◯	◯	◯	◯	◯
The online resources provided to you by NHS professionals	◯	◯	◯	◯	◯	◯
How often you saw other NHS professionals such as physiotherapists and pharmacists	◯	◯	◯	◯	◯	◯
The leaflets and booklets provided to you by NHS professionals	◯	◯	◯	◯	◯	◯
How often you saw the nursing staff	◯	◯	◯	◯	◯	◯

11. How much pain do you expect to be in for the first week after your operation?

0	1	2	3	4	5	6	7	8	9	10

No pain — Worst pain

12. How much pain do you expect to be in, for the period between 1 week and 1 month after your operation?

0	1	2	3	4	5	6	7	8	9	10

No pain — Worst pain

13. In the first week you are home after your operation, what everyday activities do you think you will require support with? Select all that apply

- ◯ Washing and bathing yourself
- ◯ Getting out of bed
- ◯ Moving around the house
- ◯ Cooking food
- ◯ Going to and using the bathroom
- ◯ Taking your medications
- ◯ None of the above
- ◯ Other - Please specify below:

14. In the period between 1 week and 1 month after your operation, what everyday activities do you think you will require support with? Select all that apply

- ○ Washing and bathing yourself
- ○ Getting out of bed
- ○ Moving around the house
- ○ Cooking food
- ○ Going to and using the bathroom
- ○ Taking your medications
- ○ None of the above
- ○ Other - Please specify below:

____

15. When do you expect the following to occur post-operatively?

	Within 1 day	*Within 3 days*	*Within 1 week*	*Within 2 weeks*	*Within 1 month*	*Within 3 months*	*More than 3 months*
Discharge from hospital	○	○	○	○	○	○	○
Be able to get up and move about	○	○	○	○	○	○	○
Use the bathroom/shower unaided	○	○	○	○	○	○	○
Be visited by the surgeon	○	○	○	○	○	○	○
Return to normal levels of physical activity	○	○	○	○	○	○	○
Return to moderate physical activities – light exercise, carrying groceries, climbing stairs, walking several blocks	○	○	○	○	○	○	○
Return to vigorous physical activities - running, heavy lifting, strenuous sports	○	○	○	○	○	○	○
Return to work	○	○	○	○	○	○	○
Resume driving	○	○	○	○	○	○	○
Post-operative pain from the operation will resolve	○	○	○	○	○	○	○
Notice an improvement in your quality of life compared to now	○	○	○	○	○	○	○

16. Since you found out you were going to have heart surgery, what lifestyle changes have you implemented? Please select all that apply

- ◯ Reduced alcohol consumption
- ◯ Reduced cigarette use
- ◯ Increased physical activity or aerobic exercise (treadmill, walking)
- ◯ Decreased physical activity or aerobic exercise (treadmill, walking)
- ◯ Improved sleep hygiene
- ◯ Improved diet
- ◯ Weight loss
- ◯ Saw medical professional to optimise medical conditions (such as diabetes, hypertension)
- ◯ Saw medical professional for mental health (anxiety, depression)
- ◯ Saw medical professional for advice about sleep, weight loss or exercise
- ◯ None of the above

17. In the last 4 weeks, how many cigarettes have you smoked per <u>day</u>?

18. <u>Before</u> your pre-assessment clinic appointment, how many cigarettes did you smoke per <u>day</u>?

19. In the last 4 weeks, how much alcohol have you consumed per <u>week</u>?

20. <u>Before</u> your pre-assessment clinic, how much alcohol did you consume per <u>week</u>?

21. Select the symptoms you have been experiencing due to your heart condition and their frequency

	All the time	*More than once a day*	*Once a day*	*More than once a week*	*Once a week or less*	*Never*
Shortness of breath	○	○	○	○	○	○
Chest pain	○	○	○	○	○	○
Dizziness	○	○	○	○	○	○
Heart palpitations (racing of heart)	○	○	○	○	○	○
Collapse/Loss of consciousness	○	○	○	○	○	○
Excessive sweating	○	○	○	○	○	○
Fatigue	○	○	○	○	○	○
Back pain	○	○	○	○	○	○
Leg swelling	○	○	○	○	○	○

22. Describe any other symptoms you have been experiencing due to your heart condition and how often you experience these

23. How many pillows do you use to sleep?

○ 0 pillows
○ 1 pillow
○ 2 pillows
○ 3 pillows
○ 4 pillows
○ 5 or more pillows

24. How many flights of stairs can you manage before feeling very short of breath?

- ◯ 0 flights of stairs
- ◯ 1 flight of stars
- ◯ 2 flights of stairs
- ◯ 3 flights of stairs
- ◯ 4 flights of stairs
- ◯ 5 flights of stairs

25. Rate the following

	Excellent	*Very Good*	*Good*	*Fair*	*Poor*	*Not applicable*
Your ability to go to and use the toilet unaided	◯	◯	◯	◯	◯	◯
Your ability to dress yourself	◯	◯	◯	◯	◯	◯
Your ability to wash yourself	◯	◯	◯	◯	◯	◯
Your ability to complete tasks at work	◯	◯	◯	◯	◯	◯
Your ability to complete tasks at home	◯	◯	◯	◯	◯	◯
Your ability to concentrate	◯	◯	◯	◯	◯	◯
Your sleep	◯	◯	◯	◯	◯	◯
Your energy levels	◯	◯	◯	◯	◯	◯

26. Compared to your health now, to what extent do you expect each of the following aspects of your health and daily life to improve or worsen following the operation?

	Much better	*Better*	*The same*	*Worse*	*Much Worse*	*Don't Know*
Your health in general	○	○	○	○	○	○
Your quality of life	○	○	○	○	○	○
Your ability to go to and use the toilet unaided	○	○	○	○	○	○
Your ability to dress yourself	○	○	○	○	○	○
Your ability to wash yourself	○	○	○	○	○	○
Your ability to care for your friends or family	○	○	○	○	○	○
Your social life	○	○	○	○	○	○
Your ability to complete tasks at work	○	○	○	○	○	○
Your ability to complete tasks at home	○	○	○	○	○	○
Your ability to perform moderate activities – light exercise, carrying groceries, climbing stairs, walking several blocks	○	○	○	○	○	○
Your ability to perform vigorous activities – running, heavy lifting, strenuous sports	○	○	○	○	○	○
Your mood	○	○	○	○	○	○
Your levels of anxiety	○	○	○	○	○	○
Your ability to concentrate	○	○	○	○	○	○
Your sleep	○	○	○	○	○	○
Your energy levels	○	○	○	○	○	○
The symptoms you are experiencing	○	○	○	○	○	○

27. What are the most important benefits you are expecting as a result of having heart surgery?

St George's University of London

St George's University Hospitals NHS
NHS Foundation Trust

		Excellent	Very good	Good	Fair	Poor
Global01	In general, would you say your health is:	☐ 5	☐ 4	☐ 3	☐ 2	☐ 1
Global02	In general, would you say your quality of life is:..	☐ 5	☐ 4	☐ 3	☐ 2	☐ 1
Global03	In general, how would you rate your physical health?	☐ 5	☐ 4	☐ 3	☐ 2	☐ 1
Global04	In general, how would you rate your mental health, including your mood and your ability to think?...	☐ 5	☐ 4	☐ 3	☐ 2	☐ 1
Global05	In general, how would you rate your satisfaction with your social activities and relationships?	☐ 5	☐ 4	☐ 3	☐ 2	☐ 1
Global09	In general, please rate how well you carry out your usual social activities and roles. (This includes activities at home, at work and in your community, and responsibilities as a parent, child, spouse, employee, friend, etc.)..................	☐ 5	☐ 4	☐ 3	☐ 2	☐ 1

		Completely	Mostly	Moderately	A little	Not at all
Global06	To what extent are you able to carry out your everyday physical activities such as walking, climbing stairs, carrying groceries, or moving a chair?...	☐ 5	☐ 4	☐ 3	☐ 2	☐ 1

In the past 7 days…

		Never	Rarely	Sometimes	Often	Always
Global10	How often have you been bothered by emotional problems such as feeling anxious, depressed or irritable?	☐ 1	☐ 2	☐ 3	☐ 4	☐ 5

		None	Mild	Moderate	Severe	Very severe
Global08	How would you rate your fatigue on average?	☐ 1	☐ 2	☐ 3	☐ 4	☐ 5

Global07	How would you rate your pain on average?..................	☐ 0 No pain	☐ 1	☐ 2	☐ 3	☐ 4	☐ 5	☐ 6	☐ 7	☐ 8	☐ 9	☐ 10 Worst imaginable pain

Figure 14. Pre-operative questionnaire

Early post-operative satisfaction and effects of cardiac surgery on quality of life

Thank you for taking part in this survey and for completing the first questionnaire before your operation. We would like to reassure you that all answers are anonymous.

This is the **early post-operative questionnaire**. You will receive the late post-operative questionnaire in 6 months' time.

In this survey we would like to find out how prepared you felt for your operation and your recovery and how you feel the recovery process has been. We would also like to explore the effects surgery has had on your quality of life.

This survey should take approximately 10 minutes to complete.

You can also complete the questionnaire online using this link:
https://forms.office.com/e/mFsevNNaYw

You can also request this online link by emailing: **angelos.anastasakis@stgeorges.nhs.uk**

What is your name? (The questionnaire will be analysed anonymously. This is so we can track who has completed each questionnaire.)

Date of Birth – dd/mm/yyyy

/ /

Date completing survey - dd/mm/yyyy

/ /

Date of operation - dd/mm/yyyy

/ /

Would you like to receive the late post-operative questionnaires in 6 months via post or email? (If you select email please provide your email address on the black line)

- ○ Post
- ○ Email: ______

St George's University of London · St George's University Hospitals NHS Foundation Trust

1. **Looking back on your operation, do you feel you were well prepared?**

- ○ Yes
- ○ Unsure
- ○ No

2. **If you answered unsure or no, why do you not feel prepared for your operation?**

3. **Having gone through with the operation, rate your understanding of the following at the time of your operation:**

	Very good	*Good*	*Neither good nor poor*	*Poor*	*Very poor*	*Not applicable*
Your heart condition	○	○	○	○	○	○
The operation you had	○	○	○	○	○	○
Risks associated with your operation	○	○	○	○	○	○
The survival rate for heart surgery	○	○	○	○	○	○
Who would be in the operating room with you	○	○	○	○	○	○
The side-effects of general anaesthetic	○	○	○	○	○	○
The support that would be available after your operation in the hospital	○	○	○	○	○	○

4. **Are there any other topics you would have liked to have been provided information on before your operation?**

5. How useful, or otherwise were the sources of information you used in hospital for information about your operation, the discharge and recovery processes?

	Extremely useful	*Very useful*	*Somewhat useful*	*Not very useful*	*Not at all useful*	*Did not use*
Leaflets/booklet from hospital	○	○	○	○	○	○
Discussion with your heart surgeon/ surgical team	○	○	○	○	○	○
Discussion with anaesthetist	○	○	○	○	○	○
Discussion with nurse	○	○	○	○	○	○
Discussion with hospital physiotherapist	○	○	○	○	○	○
Discussion with hospital pharmacist	○	○	○	○	○	○
Apps on your phone	○	○	○	○	○	○
Friends and family	○	○	○	○	○	○
Other patients	○	○	○	○	○	○
NHS Website	○	○	○	○	○	○
Other websites	○	○	○	○	○	○

6. How useful, or otherwise, are these sources of information you have been using since being discharged from hospital?

	Extremely useful	*Very useful*	*Somewhat useful*	*Not very useful*	*Not at all useful*	*Did not use*
Discussions with your GP	○	○	○	○	○	○
Discussions with your cardiologist	○	○	○	○	○	○
Discussion with your heart surgeon/ surgical team	○	○	○	○	○	○
Discussion with nurse	○	○	○	○	○	○
Leaflets/booklet from hospital	○	○	○	○	○	○
Discharge letter	○	○	○	○	○	○
Other letters	○	○	○	○	○	○
Pharmacist	○	○	○	○	○	○
Phone number in case you had any problems	○	○	○	○	○	○
Friends and family	○	○	○	○	○	○
Other patients	○	○	○	○	○	○
NHS Website	○	○	○	○	○	○
Other websites	○	○	○	○	○	○

7. After you had been discharged from hospital, how would you describe your understanding of each of the following?

	Very good	*Good*	*Neither good nor poor*	*Poor*	*Very poor*	*Not applicable*
The level of pain you could expect during recovery	○	○	○	○	○	○
When you needed to have follow-up appointments	○	○	○	○	○	○
How long to expect to be in pain for after the operation	○	○	○	○	○	○
When you could return to your usual social activities and roles	○	○	○	○	○	○
How long it would take to return to strenuous physical activity	○	○	○	○	○	○
How long it would take to return to your normal levels of physical activity	○	○	○	○	○	○
The support available to you during your recovery at home	○	○	○	○	○	○
Measures you needed to take to help your recovery	○	○	○	○	○	○
When you could resume driving	○	○	○	○	○	○
How to care for your wounds	○	○	○	○	○	○
The medication you needed to take	○	○	○	○	○	○
When you could return to work	○	○	○	○	○	○
The support you would need when engaging in everyday activities	○	○	○	○	○	○

8. What other information would you like to have been provided regarding your discharge?

9. **Below are some possible alternative ways of providing information to patients after they have been discharged from hospital. Which of the following would you have liked to have access to following your operation? Select all that apply**

○ Face-to-face meeting with the surgeon and/or the surgical team
○ Face-to-face meeting with a nurse
○ Virtual appointment with a surgeon (via telephone or video)
○ Virtual appointment with a nurse (via telephone or video)
○ Group session with other patients
○ One-to-one meeting with another patient who has had the same operation
○ App on your phone
○ Other – Please specify below:

10. How satisfied are you with the following since your operation?

	Very satisfied	*Somewhat satisfied*	*Neither satisfied nor dissatisfied*	*Somewhat dissatisfied*	*Very dissatisfied*	*Not applicable*
How often you saw your surgeon/ surgical team	○	○	○	○	○	○
Your ability to contact your surgical team or the hospital to ask any questions	○	○	○	○	○	○
How often you saw your other doctors such as your cardiologist or GP	○	○	○	○	○	○
The online resources provided to you by NHS professionals	○	○	○	○	○	○
How often you saw other NHS professionals such as physiotherapists and pharmacists	○	○	○	○	○	○
The leaflets and booklets provided to you by NHS professionals	○	○	○	○	○	○
How often you saw the nursing staff	○	○	○	○	○	○

11. How much pain were you in for the first week after your operation?

0	1	2	3	4	5	6	7	8	9	10

No pain — Worst pain

12. How much pain were you in, for the period between 1 week and 1 month after your operation?

0	1	2	3	4	5	6	7	8	9	10

No pain — Worst pain

13. In the first week you were home after your operation, what everyday activities did you require support with? Select all that apply

- ○ Washing and bathing yourself
- ○ Getting out of bed
- ○ Moving around the house
- ○ Cooking food
- ○ Going to and using the bathroom
- ○ Taking your medications
- ○ Other - Please specify below:

14. In the period between 1 week and 1 month after your operation, what everyday activities did you require support with? Select all that apply

- ◯ Washing and bathing yourself
- ◯ Getting out of bed
- ◯ Moving around the house
- ◯ Cooking food
- ◯ Going to and using the bathroom
- ◯ Taking your medications
- ◯ Other - Please specify below:

____

15. How did the level of support you required at home after your operation compare to the level you expected?

- ◯ Required much more support than expected
- ◯ Required more support than expected
- ◯ Required the amount of support expected
- ◯ Required less support than expected
- ◯ Required much less support than expected

16. When did the following occur post-operatively?

	Within 1 day	*Within 3 days*	*Within 1 week*	*Within 2 weeks*	*Within 1 month*	*Within 3 months*	*More than 3 months*
Discharge from hospital	◯	◯	◯	◯	◯	◯	◯
Be able to get up and move about	◯	◯	◯	◯	◯	◯	◯
Use the bathroom/shower unaided	◯	◯	◯	◯	◯	◯	◯
Be visited by the surgeon	◯	◯	◯	◯	◯	◯	◯
Return to normal levels of physical activity	◯	◯	◯	◯	◯	◯	◯
Return to moderate physical activities – light exercise, carrying groceries, climbing stairs, walking several blocks	◯	◯	◯	◯	◯	◯	◯
Return to vigorous physical activities - running, heavy lifting, strenuous sports	◯	◯	◯	◯	◯	◯	◯
Return to work	◯	◯	◯	◯	◯	◯	◯
Resume driving	◯	◯	◯	◯	◯	◯	◯
Post-operative pain from the operation resolved	◯	◯	◯	◯	◯	◯	◯
Notice an improvement in your quality of life compared to before	◯	◯	◯	◯	◯	◯	◯

17. Since the operation, have you developed a new shortness of breath?

- ◯ Yes
- ◯ No
- ◯ Don't know

18. If you answered yes to the previous question, is the shortness of breath worse than before your operation?

- ◯ Yes
- ◯ No
- ◯ Don't know

19. For the symptoms you were experiencing due to your heart condition, please select how often you experience them now, post-operatively

	All the time	*More than once a day*	*Once a day*	*More than once a week*	*Once a week or less*	*I no longer have this symptom*	*I never had this symptom*
Shortness of breath	◯	◯	◯	◯	◯	◯	◯
Chest pain	◯	◯	◯	◯	◯	◯	◯
Dizziness	◯	◯	◯	◯	◯	◯	◯
Heart palpitations (racing of heart)	◯	◯	◯	◯	◯	◯	◯
Collapse/Loss of consciousness	◯	◯	◯	◯	◯	◯	◯
Excessive sweating	◯	◯	◯	◯	◯	◯	◯
Fatigue	◯	◯	◯	◯	◯	◯	◯
Back pain	◯	◯	◯	◯	◯	◯	◯
Leg swelling	◯	◯	◯	◯	◯	◯	◯

20. Are there any other symptoms you were experiencing due to your heart condition. Please mention how often you experience these symptoms now post-operatively.

21. How many pillows do you use to sleep now?

- ○ 0 pillows
- ○ 1 pillow
- ○ 2 pillows
- ○ 3 pillows
- ○ 4 pillows
- ○ 5 or more pillows

22. How many flights of stairs can you manage before feeling very short of breath now?

- ○ 0 flights of stairs
- ○ 1 flight of stars
- ○ 2 flights of stairs
- ○ 3 flights of stairs
- ○ 4 flights of stairs
- ○ 5 or more flights of stairs

23. Rate the following

	Excellent	*Very Good*	*Good*	*Fair*	*Poor*	*Not applicable*
Your ability to go to and use the toilet unaided	○	○	○	○	○	○
Your ability to dress yourself	○	○	○	○	○	○
Your ability to wash yourself	○	○	○	○	○	○
Your ability to complete tasks at work	○	○	○	○	○	○
Your ability to complete tasks at home	○	○	○	○	○	○
Your ability to concentrate	○	○	○	○	○	○
Your sleep	○	○	○	○	○	○
Your energy levels	○	○	○	○	○	○

24. What are the most important benefits you have experienced as a result of having heart surgery?

25. What are the most important negative effects of heart surgery you have experienced since your operation?

26. How satisfied are you with each of the following?

	Very satisfied	*Somewhat satisfied*	*Neither satisfied nor dissatisfied*	*Somewhat dissatisfied*	*Very dissatisfied*	*Not applica ble*
How your pain was controlled in hospital	○	○	○	○	○	○
How your pain was controlled when you returned home after surgery	○	○	○	○	○	○
How long it took to return to your expected levels of daily activity	○	○	○	○	○	○
How long it took to return to work	○	○	○	○	○	○
Overall outcome of your surgery	○	○	○	○	○	○

St George's University of London — St George's University Hospitals NHS Foundation Trust

		Excellent	Very good	Good	Fair	Poor
Global01	In general, would you say your health is:	☐ 5	☐ 4	☐ 3	☐ 2	☐ 1
Global02	In general, would you say your quality of life is:...	☐ 5	☐ 4	☐ 3	☐ 2	☐ 1
Global03	In general, how would you rate your physical health?	☐ 5	☐ 4	☐ 3	☐ 2	☐ 1
Global04	In general, how would you rate your mental health, including your mood and your ability to think?...	☐ 5	☐ 4	☐ 3	☐ 2	☐ 1
Global05	In general, how would you rate your satisfaction with your social activities and relationships?	☐ 5	☐ 4	☐ 3	☐ 2	☐ 1
Global09	In general, please rate how well you carry out your usual social activities and roles. (This includes activities at home, at work and in your community, and responsibilities as a parent, child, spouse, employee, friend, etc.)...................	☐ 5	☐ 4	☐ 3	☐ 2	☐ 1

		Completely	Mostly	Moderately	A little	Not at all
Global06	To what extent are you able to carry out your everyday physical activities such as walking, climbing stairs, carrying groceries, or moving a chair?...	☐ 5	☐ 4	☐ 3	☐ 2	☐ 1

In the past 7 days…

		Never	Rarely	Sometimes	Often	Always
Global10	How often have you been bothered by emotional problems such as feeling anxious, depressed or irritable?	☐ 1	☐ 2	☐ 3	☐ 4	☐ 5

		None	Mild	Moderate	Severe	Very severe
Global08	How would you rate your fatigue on average?	☐ 1	☐ 2	☐ 3	☐ 4	☐ 5

Global07	How would you rate your pain on average?..................	☐ 0 No pain	☐ 1	☐ 2	☐ 3	☐ 4	☐ 5	☐ 6	☐ 7	☐ 8	☐ 9	☐ 10 Worst imaginable pain

Figure 15. Early post-operative questionnaire

St George's University of London — St George's University Hospitals NHS Foundation Trust

Late post-operative satisfaction and effects of cardiac surgery on quality of life

Thank you for completing the first and second questionnaires.

This is the **late post-operative questionnaire.** This is the final survey you will receive for this research project.

In this survey we would like to find out how the recovery process has been for you, the effects that surgery has had on your quality of life and how satisfied you are with the outcomes of your surgery.

This survey should take approximately 10 minutes to complete

You can also complete the questionnaire online using this link:

https://forms.office.com/e/4uUq9cu7jF

You can also request this online link by emailing: **angelos.anastasakis@stgeorges.nhs.uk**

What is your name? (The questionnaire will be analysed anonymously. This is so we can track who has completed each questionnaire.)

Date of Birth – dd/mm/yyyy

/ /

Date completing survey - dd/mm/yyyy

/ /

Date of operation - dd/mm/yyyy

/ /

Have you completed the pre-operative and early post-operative surveys?

○ Yes
○ No

1. **How useful, or otherwise, are these sources of information you have been using since being discharged from hospital?**

	Extremely useful	*Very useful*	*Somewhat useful*	*Not very useful*	*Not at all useful*	*Did not use*
Discussions with your GP	○	○	○	○	○	○
Discussions with your cardiologist	○	○	○	○	○	○
Discussion with your heart surgeon/ surgical team	○	○	○	○	○	○
Discussion with nurse	○	○	○	○	○	○
Leaflets/booklet from hospital	○	○	○	○	○	○
Discharge letter	○	○	○	○	○	○
Other letters	○	○	○	○	○	○
Pharmacist	○	○	○	○	○	○
Phone number in case you had any problems	○	○	○	○	○	○
Friends and family	○	○	○	○	○	○
Other patients	○	○	○	○	○	○
NHS Website	○	○	○	○	○	○
Other websites	○	○	○	○	○	○

2. **How satisfied are you with the following since your operation?**

	Very satisfied	*Somewhat satisfied*	*Neither satisfied nor dissatisfied*	*Somewhat dissatisfied*	*Very dissatisfied*	*Not applica ble*
How often you saw your surgeon/ surgical team	○	○	○	○	○	○
Your ability to contact your surgical team or the hospital to ask any questions	○	○	○	○	○	○
How often you saw your other doctors such as your cardiologist or GP	○	○	○	○	○	○
The online resources provided to you by NHS professionals	○	○	○	○	○	○
How often you saw other NHS professionals such as physiotherapists and pharmacists	○	○	○	○	○	○

	Very satisfied	*Somewhat satisfied*	*Neither satisfied nor dissatisfied*	*Somewhat dissatisfied*	*Very dissatisfied*	*Not applica ble*
The leaflets and booklets provided to you by NHS professionals	○	○	○	○	○	○
How often you saw the nursing staff	○	○	○	○	○	○

3. When did the following occur post-operatively?

	Within 1 week	*Within 2 weeks*	*Within 1 month*	*Within 2 months*	*Within 3 months*	*Within 6 months*	*More than 6 months*
Be able to get up and move about	○	○	○	○	○	○	○
Use the bathroom/shower unaided	○	○	○	○	○	○	○
Return to normal levels of physical activity	○	○	○	○	○	○	○
Return to moderate physical activities – light exercise, carrying groceries, climbing stairs, walking several blocks	○	○	○	○	○	○	○
Return to vigorous physical activities - running, heavy lifting, strenuous sports	○	○	○	○	○	○	○
Return to work	○	○	○	○	○	○	○
Resume driving	○	○	○	○	○	○	○
Post-operative pain from the operation resolved	○	○	○	○	○	○	○
Notice an improvement in your quality of life compared to before	○	○	○	○	○	○	○

4. Since the operation, have you developed a new shortness of breath?

- ○ Yes
- ○ No
- ○ Don't know

5. If you answered yes to the previous question, is the shortness of breath worse than before your operation?

- ○ Yes
- ○ No
- ○ Don't know

6. For the symptoms you were experiencing due to your heart condition, please select how often you experience them now, post-operatively

	All the time	*More than once a day*	*Once a day*	*More than once a week*	*Once a week or less*	*I no longer have this symptom*	*I never had this symptom*
Shortness of breath	○	○	○	○	○	○	○
Chest pain	○	○	○	○	○	○	○
Dizziness	○	○	○	○	○	○	○
Heart palpitations (racing of heart)	○	○	○	○	○	○	○
Collapse/Loss of consciousness	○	○	○	○	○	○	○
Excessive sweating	○	○	○	○	○	○	○
Fatigue	○	○	○	○	○	○	○
Back pain	○	○	○	○	○	○	○
Leg swelling	○	○	○	○	○	○	○

7. Are there any other symptoms you were experiencing due to your heart condition. Please mention how often you experience these symptoms now post-operatively.

8. How many pillows do you use to sleep now?

- ◯ 0 pillows
- ◯ 1 pillow
- ◯ 2 pillows
- ◯ 3 pillows
- ◯ 4 pillows
- ◯ 5 or more pillows

9. How many flights of stairs can you manage before feeling very short of breath now?

- ◯ 0 flights of stairs
- ◯ 1 flight of stars
- ◯ 2 flights of stairs
- ◯ 3 flights of stairs
- ◯ 4 flights of stairs
- ◯ 5 or more flights of stairs

10. Rate the following

	Excellent	*Very Good*	*Good*	*Fair*	*Poor*	*Not applicable*
Your ability to go to and use the toilet unaided	◯	◯	◯	◯	◯	◯
Your ability to dress yourself	◯	◯	◯	◯	◯	◯
Your ability to wash yourself	◯	◯	◯	◯	◯	◯
Your ability to complete tasks at work	◯	◯	◯	◯	◯	◯
Your ability to complete tasks at home	◯	◯	◯	◯	◯	◯
Your ability to concentrate	◯	◯	◯	◯	◯	◯
Your sleep	◯	◯	◯	◯	◯	◯
Your energy levels	◯	◯	◯	◯	◯	◯

11. What are the most important benefits you have experienced as a result of having heart surgery?

12. What are the most important negative effects of heart surgery you have experienced since your operation?

13. How satisfied are you with each of the following?

	Very satisfied	*Somewhat satisfied*	*Neither satisfied nor dissatisfied*	*Somewhat dissatisfied*	*Very dissatisfied*	*Not applicable*
How long it took for you to return to your daily activities (e.g. housework, social activities)	○	○	○	○	○	○
How long it took to return to work	○	○	○	○	○	○
How long it took to return to your normal exercise routine	○	○	○	○	○	○
The appearance of your scars	○	○	○	○	○	○
Overall outcome of your surgery	○	○	○	○	○	○

14. Looking back, if you "had to do it all over again," would you have the surgery again?

- ○ Yes
- ○ Maybe (probably yes)
- ○ Unsure
- ○ Don't think so
- ○ Never

15. Would you recommend this surgery to someone else?

- ○ Yes
- ○ Maybe (probably yes)
- ○ Unsure
- ○ Don't think so
- ○ Never

16. What advice would you give patients who might undergo the same operation as you in the future?

17. How much better or worse is your quality-of-life following heart surgery compared to what you expected?

- ○ Much better than expected
- ○ Better than expected
- ○ Neither better nor worse than expected
- ○ Worse than expected
- ○ Much worse than expected

St George's University of London — St George's University Hospitals NHS Foundation Trust

		Excellent	Very good	Good	Fair	Poor
Global01	In general, would you say your health is:	☐ 5	☐ 4	☐ 3	☐ 2	☐ 1
Global02	In general, would you say your quality of life is:..	☐ 5	☐ 4	☐ 3	☐ 2	☐ 1
Global03	In general, how would you rate your physical health?	☐ 5	☐ 4	☐ 3	☐ 2	☐ 1
Global04	In general, how would you rate your mental health, including your mood and your ability to think?..	☐ 5	☐ 4	☐ 3	☐ 2	☐ 1
Global05	In general, how would you rate your satisfaction with your social activities and relationships?	☐ 5	☐ 4	☐ 3	☐ 2	☐ 1
Global09	In general, please rate how well you carry out your usual social activities and roles. (This includes activities at home, at work and in your community, and responsibilities as a parent, child, spouse, employee, friend, etc.)..................	☐ 5	☐ 4	☐ 3	☐ 2	☐ 1

		Completely	Mostly	Moderately	A little	Not at all
Global06	To what extent are you able to carry out your everyday physical activities such as walking, climbing stairs, carrying groceries, or moving a chair?..	☐ 5	☐ 4	☐ 3	☐ 2	☐ 1

In the past 7 days…

		Never	Rarely	Sometimes	Often	Always
Global10	How often have you been bothered by emotional problems such as feeling anxious, depressed or irritable?	☐ 1	☐ 2	☐ 3	☐ 4	☐ 5

		None	Mild	Moderate	Severe	Very severe
Global08	How would you rate your fatigue on average?	☐ 1	☐ 2	☐ 3	☐ 4	☐ 5

Global07	How would you rate your pain on average?...................	☐ 0 No pain	☐ 1	☐ 2	☐ 3	☐ 4	☐ 5	☐ 6	☐ 7	☐ 8	☐ 9	☐ 10 Worst imaginable pain

Figure 16. Late post-operative questionnaire
