# Supplementary material for: Patients’ Experience With Enhanced Recovery After Cardiac Surgery
Source: Interdiscip Cardiovasc Thorac Surg. 2026 Mar 20;41(3):ivag051. doi: 10.1093/icvts/ivag051 (PMC13006205; doi:10.1093/icvts/ivag051)
Supplement: ivag051_Supplementary_Data [file ivag051_supplementary_data.zip › Supplementary material 1 - Tables and Figures.docx]

| Supplementary Table S1: Cardiac ERAS protocol utilised in this study. For the study duration green denotes compliance ≥90%, yellow 80 - 90% and orange 70-80% | | |
| --- | --- | --- |
| Pre-operative | **Intra-operative** | **Post-operative** |
| Screening and stopping smoking and hazardous alcohol consumption 4 weeks before elective surgery | Administration of intravenous cephalosporin prophylactic antibiotic 30-60 min prior to surgery | Goal-directed fluid therapy |
| Screening for frailty | Clipping immediately prior to surgery | Opioid-sparing pain management plan |
| Prehabilitation for patients undergoing elective surgery | Use a chlorhexidine-alcohol–based solution for skin preparation before surgery | Avoidance of persistent hypothermia (≤36°C) after cardiopulmonary bypass in the early postoperative period |
| Promotion of education utilising patient engagement tools | Avoidance of hyperthermia (>37.9°C) while rewarming on CPB | Maintenance of chest tube patency to prevent retained blood without stripping or breaking the sterile field |
| Pre-operative measurement of haemoglobin A1c | The use of tranexamic acid for on-pump cardiac surgical procedures | Postoperative systematic delirium screening tool use at least once per nursing shift |
| Pre-operative measurement of albumin and correction of nutritional deficiency | Use of endoscopic conduit harvest for CABG | Use of digital chest drainage system |
| Pre-operative screening for anaemia and treatment with IV or oral iron |  | An insulin infusion to treat hyperglycaemia in all patients postoperatively |
| Continued consumption of clear fluids up until two to four hours before induction of general anaesthesia |  | Strategies to promote extubation within 6 h of surgery when appropriate |
| Pre-operative carbohydrate loading |  | Removal of operative wound dressing after 48 hours |
| Topical intranasal decolonization prior to surgery |  | Chemical or mechanical thromboprophylaxis after surgery |
| Accelerated pre-operative ERAS bundle for patients undergoing urgent surgery |  |  |

**Supplementary Table S2: Cardiac ERAS protocol utilised in this study**

| **Criteria for Post Operative Discharge of CTICU Patients to CTS Ward** |
| --- |
| Reviewed by Consultant Intensivist and discharge agreed |
| Reviewed by surgical team and ward nursing team and discharge agreed |
| Extubated and airway safe |
| RR 12 -20 |
| Able to cough and clear secretions ( without adjuncts) |
| PaO2 > 9 KPa with FiO2 < 40% or Sats > 94% ( or 88- 92% for COPD patients) |
| PaCO2 3.4 – 6.5 KPa (or at pre op baseline if outside this range) |
| No CPAP or NIV (unless resumption of normal Home Nocturnal CPAP which patient can manage independently) |
| Systolic BP within prescribed parameters/ within 20% of preop BP |
| No vasoactive medications |
| Not dependent on epicardical pacing : intrinsic rate must be > 40 |
| Stable Rhythm ( Atrial Fibrillation not a contraindication if rate controlled and haemodynamically stable) |
| Post operative ECGs reviewed and documented in medical discharge summary |
| Temperature > 36 < 38 degrees |
| All blood results reviewed, within normal range or down trending including U & Es and CRP |
| Hb > 75g/dl |
| Lactate < 2 mmols/L: trend stable and improving |
| Patient alert, orientated and calm, equal power in all limbs and PEARL or back to pre op baseline or recovering from identified and managed new stroke |
| Pain well controlled/ able to deep breathe and cough |
| Blood Glucose controlled within prescribed range 6-10mmols/L (ward accept insulin infusion) |
| Drain output < 50mls/ hr for preceding 6 hours |
| Urine Output > 0.5 mls/kg/hr |
| **If all the above criteria are met, the patient can be transferred to the Ward within 4 hours of fullfilling these criteria** |
| **Criteria for Post Operative Discharge of CTICU Patients to CTS HDU** |
| Reviewed by Consultant Intensivist and discharge agreed |
| Reviewed by surgical team and HDU nursing team and discharge agreed |
| **Optiflow** maximum of 40% with up to 60 L/min 02 for 6 hours |
| No NIV or CPAP |
| **Noradrenaline** permitted to a maximum of 0.1 mcg/kg/min and not escalating in the last 3 hours (**EXCEPT** Friday discharges with Noradrenaline permitted up to a maximum of 0.05mcg/kg/min for at least 3 hours and a with a reducing trajectory) |
| Patients can be pacing dependent due to bradycardia < 40 bpm or asystole. |
| Chest drain Output < 50 mls/hr for at least 2 hours |
| Patient reasonably expected to be able to be stepped down to the CTS ward by the time of any planned closure of HDU |
| HDU accept arterial lines, CVC, pacing wires, paced patients and chest drains |
| HDU NIC will assess if the patient is suitable for HDU, particularly in relation to delirium/ confusion and overnight requirements for additional sedatives eg: haloperidol. Patients will be assessed in relation to the current work load and patient group in HDU. |
| No Mechanical Circulatory Support ie: IABP, Impella, ECMO, VAD |
| No patient requiring isolation in a side room for infection reasons |

**
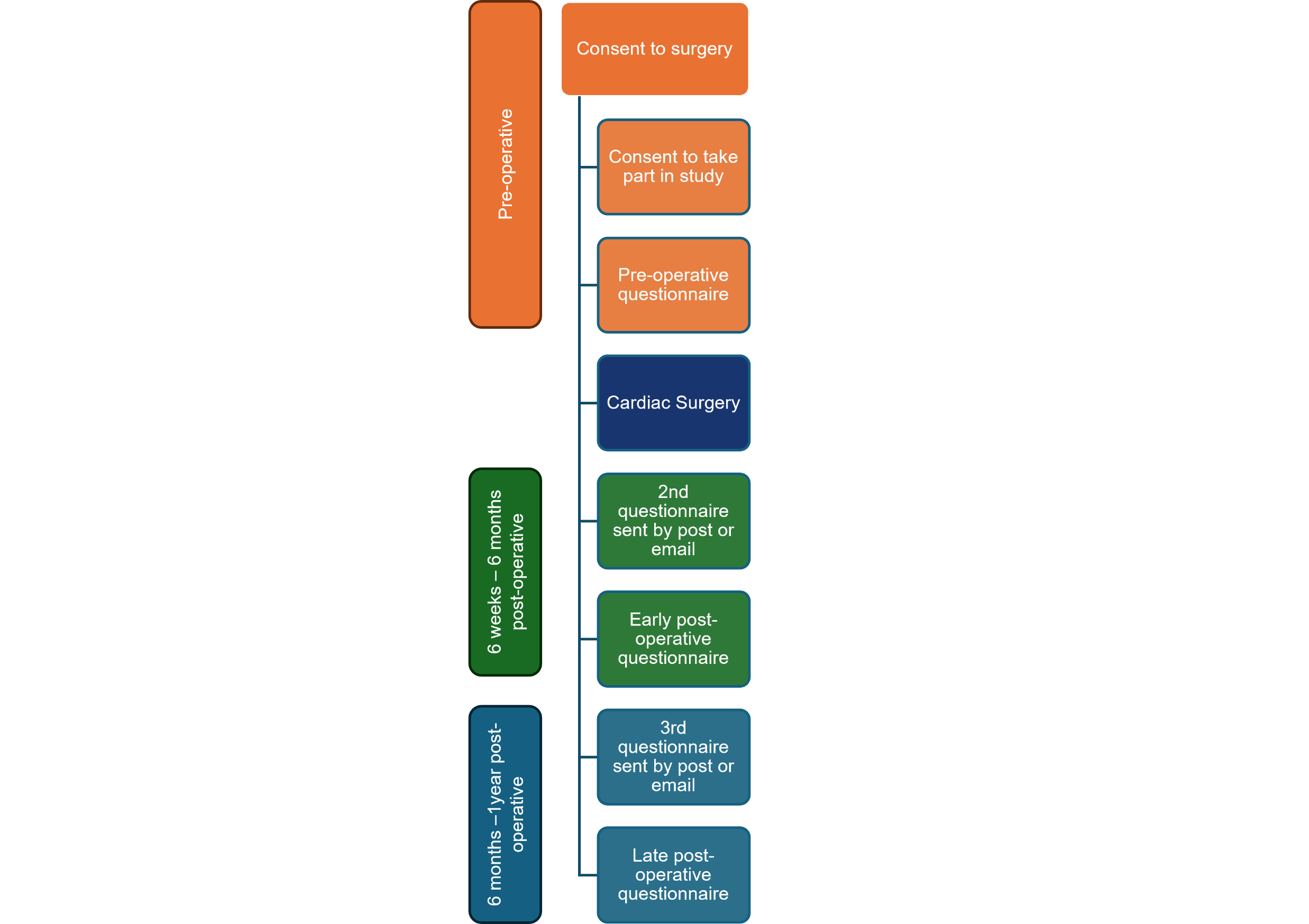
**

**Supplementary Figure S1.** Study Flowchart


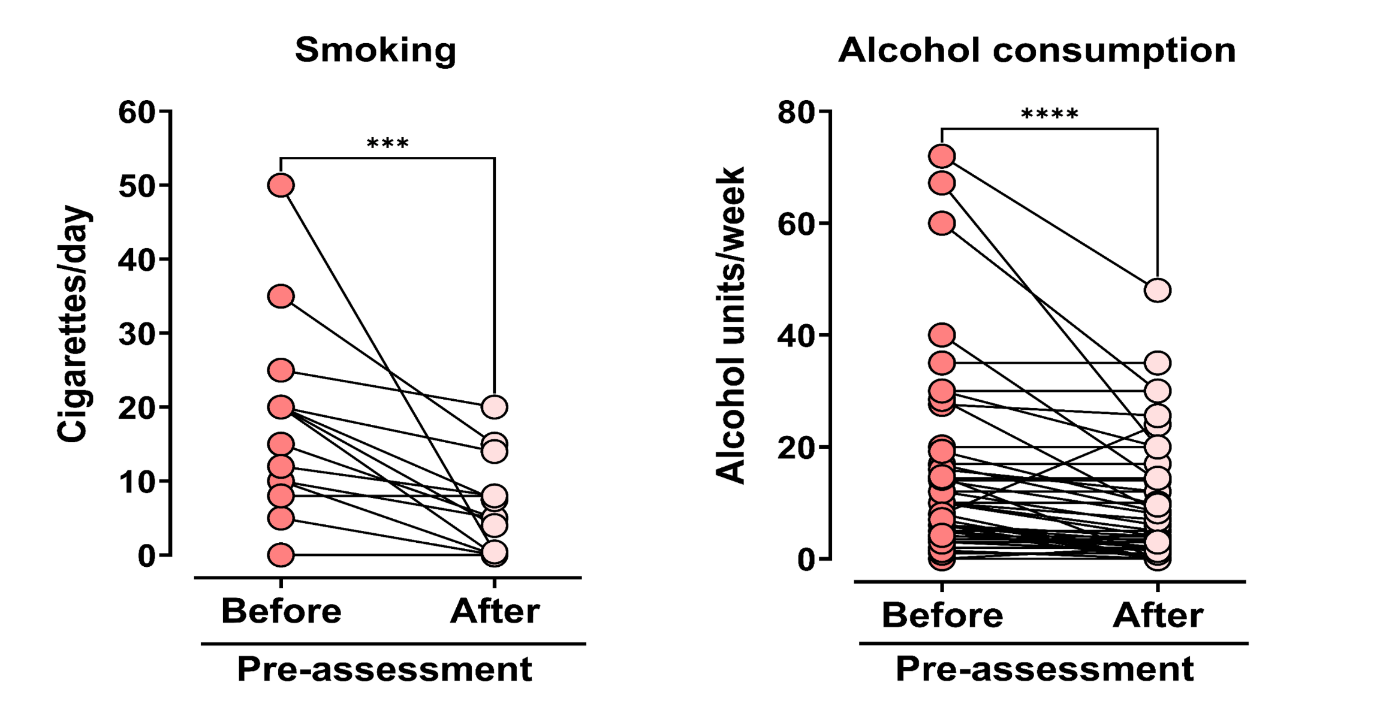


**Supplementary Figure S2.** Cigarette consumption/day and units of alcohol consumption before (at the time of pre-assessment clinic) and after (at the time of cardiac surgery) cardiac pre-assessment clinic in elective patients (N=89).

**
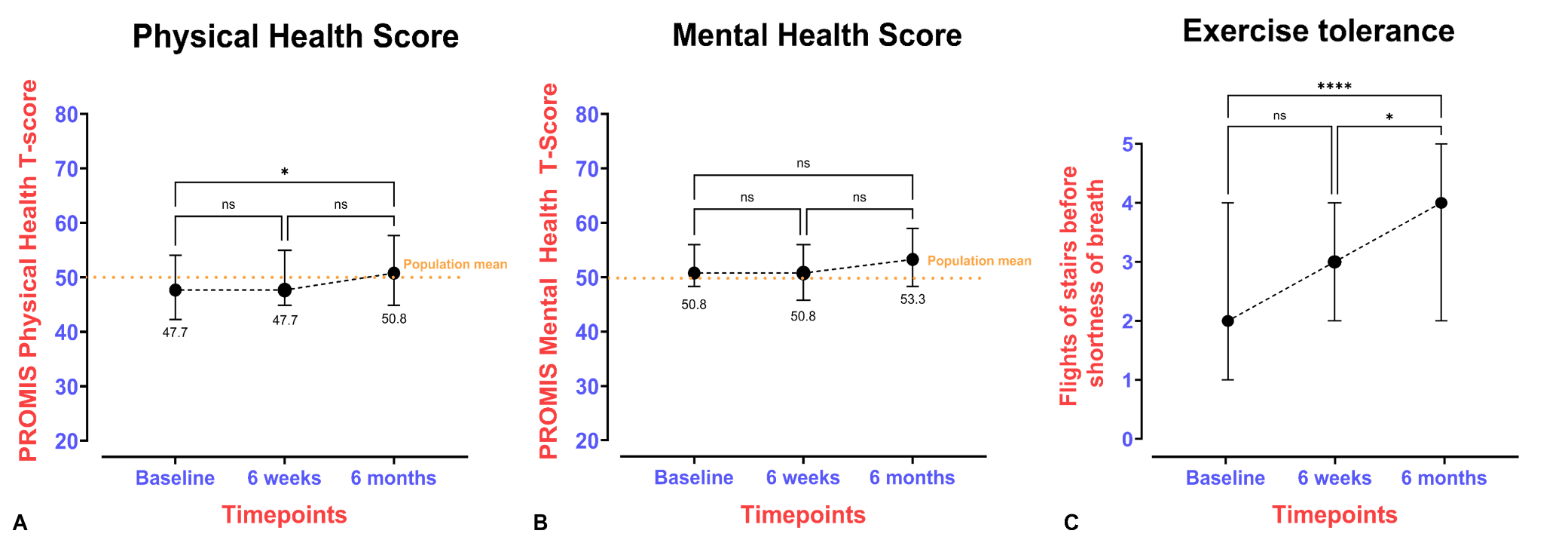
**

**Supplementary Figure S3.** Quality of life and exercise tolerance at baseline, 6 weeks and 6 months post operatively. Quality of life assessed using PROMIS global health measure v1.2. *P<0.05 for within group comparison carried out using the Friedman test and corrected using Dunn’s multiple comparisons test. (N=109)

**
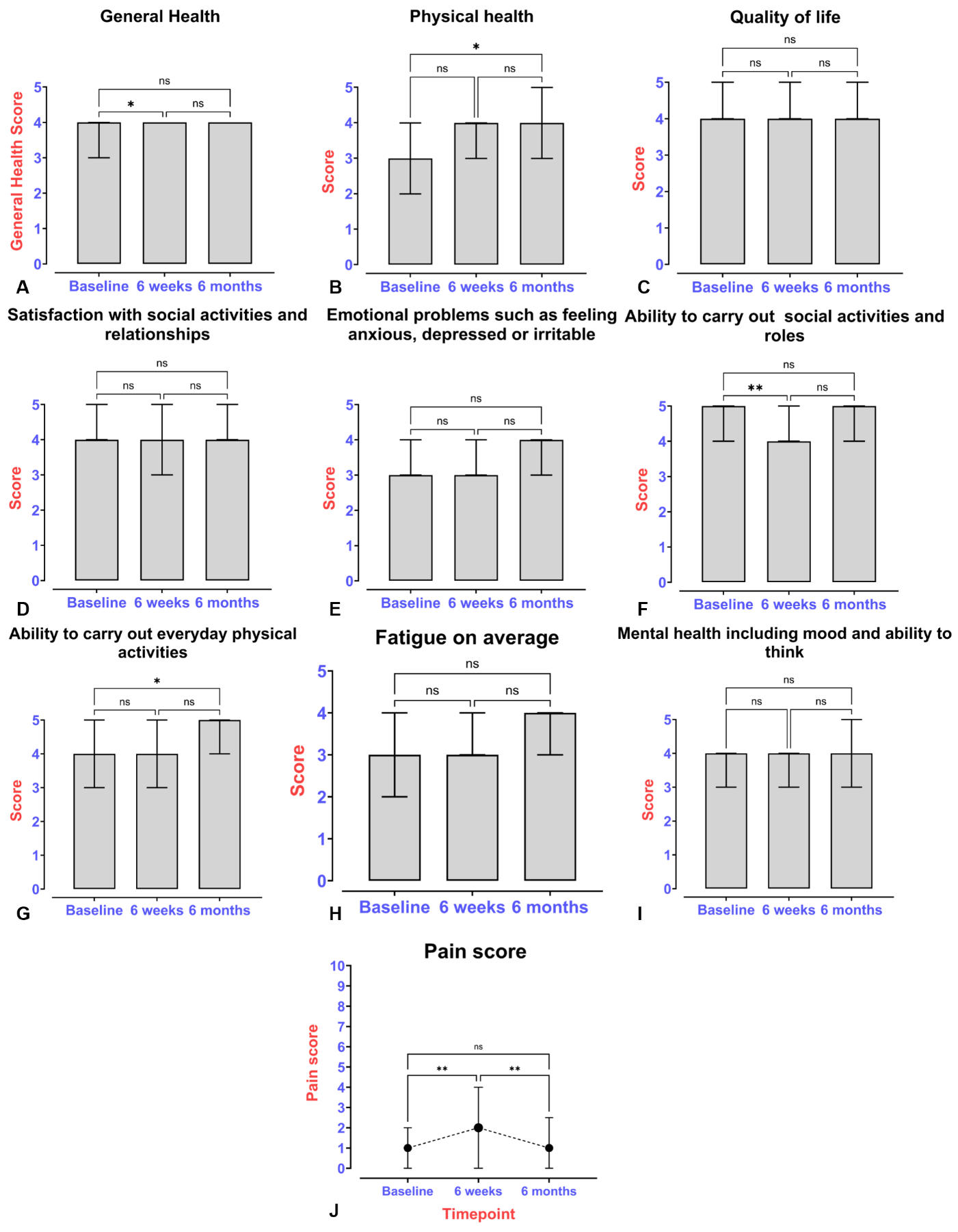
**

**Supplementary Figure S4.** Individual components of the PROMIS global health measure v1.2. *P<0.05, **P<0.01 for within group comparison carried out using the Friedman test and corrected using Dunn’s multiple comparisons test. (N=109)
